# Potent immunomodulatory and antitumor effect of anti-CD20-IL2no-alpha tri-functional immunocytokine for cancer therapy

**DOI:** 10.3389/fimmu.2022.1021828

**Published:** 2022-12-09

**Authors:** Ana Victoria Casadesús, Beatriz María Cruz, Wilden Díaz, Miguel Ángel González, Tania Gómez, Briandy Fernández, Addys González, Nuris Ledón, Katya Sosa, Kathleen Castro, Armando López, Claudia Plasencia, Yaima Ramírez, Jean-Luc Teillaud, Calixto Hernández, Kalet León, Tays Hernández

**Affiliations:** ^1^ Department of Chimeric Proteins, Immunobiology Division, Center of Molecular Immunology (CIM), Havana, Cuba; ^2^ Quality Control Division, Center of Molecular Immunology (CIM), Havana, Cuba; ^3^ Department of Animal Facilities, Immunobiology Division, Center of Molecular Immunology (CIM), Havana, Cuba; ^4^ Department of Innovation´s Management, Center of Molecular Immunology (CIM), Havana, Cuba; ^5^ Development Division, Center of Molecular Immunology (CIM), Havana, Cuba; ^6^ Laboratory of Immune Microenvironment and Immunotherapy, Centre d’immunologie et des maladies infectieuses (CIMI-Paris), Inserm UMRS1135, Sorbonne University, Paris, France; ^7^ Hematology Department, Hermanos Ameijeiras Hospital, Havana, Cuba; ^8^ Research Division, Center of Molecular Immunology (CIM), Havana, Cuba

**Keywords:** anti-CD20, immunocytokine, IL-2 mutein, immunotherapy, lymphoma

## Abstract

**Introduction:**

The anti-CD20 antibody rituximab (RTX) has substantially improved outcomes of patients with B-cell lymphomas, although more efficient therapies are needed for refractory or relapsing lymphomas. An approach to increase the clinical effectiveness of anti-tumor therapy is the use of antibody-cytokine fusion proteins (immunocytokines (ICKs)) to deliver at the tumor site the antibody effector functions and cytokines that trigger anti-tumor activities. In particular, IL-2-based ICKs have shown significant results in preclinical studies but not in clinical trials due to the toxicity profile associated to high doses IL-2 and the undesired expansion of Tregs.

**Methods:**

To improve the efficacy of RTX therapy, we fused a murine (mIgG2a) or a human (hIgG1) version of RTX to a mutated IL-2 (no-alpha mutein), which has a disrupted affinity for the high affinity IL-2 receptor (IL-2R) to prevent the stimulation of Tregs and reduce the binding to endothelial cells expressing CD25, the α chain of high affinity IL-2R. Characterization of anti-CD20-IL2no-alpha ICKs was performed by SDS-PAGE, Western-blotting and SEC-HPLC and also by several functional *in vitro* techniques like T-cell proliferation assays, apoptosis, CDC and ADCC assays. The *in vivo* activity was assessed by using murine tumor cells expressing huCD20 in C57/Bl6 mice.

**Results:**

Both ICKs exhibited similar *in vitro* specific activity of their IL2no-alpha mutein moieties and kept CD20-binding capacity. Anti-CD20-IL2no-alpha (hIgG1) retained antibody effector functions as complement-dependent cytotoxicity and enhanced direct apoptosis, NK cell activation and antibody-dependent cellular cytotoxicity relative to RTX. In addition, both ICKs demonstrated a higher antitumor efficacy than parental molecules or their combination in an EL4-huCD20 tumor model in immunocompetent mice. Anti-CD20-IL2no-alpha (hIgG1) strongly expanded NK and CD8+ T cells but not Tregs in tumor-bearing mice.

**Discussion:**

These findings suggest that anti-CD20-IL2no-alpha could represent an alternative treatment for B cell lymphoma patients, mainly those refractory to RTX therapy.

## Introduction

Rituximab (RTX) has substantially improved treatment outcomes in B-cell non-Hodgkin lymphomas (B-NHLs), achieving high response rates in low-grade B-cell lymphomas and increasing survival in both indolent and aggressive forms when combined with chemotherapy (1). As RTX is an anti-CD20 type I antibody, it is able to translocate CD20 to lipid rafts, preferentially activate complement-dependent cytotoxicity (CDC) and antibody-dependent cell-mediated cytotoxicity (ADCC) and induce a caspase-dependent apoptosis, mechanisms that support its therapeutic effect (1, 2).

Thus, new approaches have been developed to ameliorate anti-CD20 efficacy and overcome RTX resistance. Some of them involve combination with radioimmunotherapy, the generation of novel antibodies or antibody formats, and combination or fusion to immunostimulatory cytokines that potentiate its effector functions (3–5). In this sense, anti-CD20-cytokine fusion proteins or anti-CD20 ICKs have emerged as next generation molecules aimed at delivering higher amounts of cytokines at the tumor site while recruiting classical antibody activities (6–11). IL-2 has been explored as an attractive candidate for generating ICKs due to its potent capacity to induce the proliferation and the cytotoxic activity of T cells (12) and to enhance ADCC mediated by natural killer (NK) cells (13). In 2005, Gillies et al. (7) produced an anti-CD20-IL2(DI-Leu16-IL-2), based on the fusion of IL-2 to a deimmunized anti-CD20 antibody. This ICK retained full anti-CD20 activity and exhibited an enhanced ADCC relative to the parental anti-CD20 antibody. DI-Leu16-IL-2 was far more effective against human CD20+ lymphoma cells in immunodeficient mice than 25-fold higher doses of anti-CD20 mAb plus IL-2 (7). In the recent years, other attempts have followed the idea of developing anti-CD20-IL2 ICKs. Marusic et al. (2016) (9) reported the first example of a scFv-Fc-engineered recombinant ICK based on the therapeutic RTX antibody scaffold and IL-2, which was assembled in plants. This molecule was able to bind CD20 molecule and elicited ADCC by human PBMC against Daudi cells while showing IL-2 activity in proliferation assays.

In general, those ICKs based on IL-2 have shown inadequate pharmacokinetic properties and a toxicity profile similar to aldesleukin (14–16). Particularly, DI-Leu16-IL-2 showed promising results in Phase 2 clinical trials, but with some adverse effects and expansion of Tregs (15, 16). Although conflicting results have been reported regarding the prognostic significance of Treg infiltration in NHL (17), several studies have shown a direct correlation between Tregs and bad prognosis in these lymphomas (18–21).

Novel attempts to improve the anti-tumor potential of IL-2 in the clinics have been recently made. They include re-engineering IL-2 to bind to different IL-2 receptor conformations, the use of PEGylated IL-2 agonists and IL-2/mAb complexes, that selectively improve IL-2 response by specifically-targeted immune cell populations (22). In line with this, different groups developing IL-2-bearing ICKs have focused on using mutated versions of IL-2 in a scaffold mostly based on cytokine monovalent formats and full-length IgG devoid of Fc-mediated effector functions.

Cergutuzumab amunaleukin (CEA-IL2v, RG7813), an example of this approach, comprises an engineered variant of IL-2, designed to abolish CD25 binding. CD25 is the α chain of the IL-2 receptor (IL-2R). Its association with the β and γ chains of the intermediate affinity IL-2R (βγ IL-2R) gives the receptor (αβγ IL-2R) a high affinity for IL-2. The latter IL-2Rαβγ receptor is predominantly expressed by Tregs while the βγIL-2R is mostly expressed by CD8+ T cells and NK cells. This ICK demonstrated improved tolerability, pharmacokinetics and tumor targeting as well as preferential activation of CD8+ and NK immune effector cells over Tregs as compared with its wild-type IL-2-based counterpart (14). These attributes were also considered in the design and development of the ICKs anti-PD1-IL2v (23) and FAP-IL2v (4). Despite the evidence in the clinics showing less toxicity and Treg expansion, some IL-2 no alpha chain- reactivity based ICK failed to prove efficacy as monotherapy or even in combination with checkpoint inhibitors, which indicates the need to explore new strategies around this concept (22). Another study with a super mutant IL-2 (sumIL-2) with decreased CD25 binding and increased CD122 (βIL-2R) binding, fused to a tumor-targeting antibody, revealed the therapeutic potential of antibody-cytokine fusion proteins comprising next-generation IL-2. This ICK led not only to reduced toxicity but also had an enhanced effect on the targeting of CD8+ T cells inside tumor tissue (24).

Some years ago, a mutated IL-2 (termed no-alpha mutein) was developed which has a disrupted affinity for the high affinity IL-2R, expands preferentially CD8+ T lymphocytes and NK cells over Tregs (25, 26) and exerts a higher anti-metastatic effect than IL-2 in 3LL-D122 and B16 tumor models (25). Of note, it also displays a reduced toxicity as compared to its wild-type counterpart. In a previous work, we demonstrated that anti-CD20 antibody administered in combination with no-alpha mutein augments the survival rate of immunocompetent mice challenged with EL4-huCD20 cells relative to animals treated with anti-CD20 ± native r-IL-2. Moreover, the combination with no-alpha mutein, but not IL-2, induced an expansion of granzyme B+/perforin+ cells in splenic NK and CD8+ T cell compartments, a reduction of Tregs and an increase in activated macrophages (27). These results make this IL-2 variant an appealing partner to be fused to anti-CD20 antibodies.

In the present study, we generated the first anti-CD20 ICK based on a rationally engineered IL-2 (no-alpha mutein). This RTX-derived fusion protein adopts a tri-functional format. This format 1) binds to CD20, with the ability to trigger cell apoptosis as does RTX, 2) binds to FcγR, with the ability to trigger ADCC, and 3) selectively engages βγ IL-2R through its mutated IL-2 moiety, leading to the activation of cells expressing the latter receptor such as NK cells. Here, we describe its biological properties *in vitro*, its capacity of expanding NK and CD8+ T cells but not Tregs in a tumoral setting, and its high anti-tumor potency *in vivo*, highlighting its potential clinical benefit in B-cell malignancies.

## Materials and methods

### Cell lines

The human Burkitt’s lymphoma Ramos and Raji cell lines, the T lymphocyte Jurkat cell line, the human embryonic kidney 293T cells (HEK-293T), the murine EL4 cells and Chinese hamster ovary (CHO-K1) cells were purchased from American Type Culture Collection. The mouse thymoma EL4 cells expressing human CD20 (EL4-huCD20) were kindly provided by J. Golay (Ospedali Riunti di Bergamo, Bergamo, Italy). These cells are CD25^-^ CD4^-^ CD3^+^. The murine T cell line CTLL-2 was donated by Dr. A. Santos (Center of Genetic Engineering and Biotechnology, La Habana, Cuba). All cells were grown at 37°C under a humidified 5% CO_2_ atmosphere.

### Immunocytokines, antibodies and cytokines

The ICKs and antibodies were affinity-purified from culture supernatants by using a MabSelect Protein A column (GE Healthcare, Sweden) and size exclusion/exchange chromatography. ICK and antibodies concentration was determined by measuring the absorbance at 280nm with a molar extinction coefficient of 1.431 and purity was analyzed in non-reducing conditions on SDS-PAGE, and on an analytical Superdex S-200 size exclusion column (GE Healthcare, Sweden).

Rituximab, a chimeric mAb that recognizes human CD20 molecule, was purchased from Roche. MOPC-173 mAb (Abcam) was used as isotype-matched control. The no-alpha mutein was produced and purified at CIM as previously described (25). The human IL-2 was purchased from Peprotech. Of note, human IL-2 (hIL-2) binds to mouse βγ and αβγ IL-2Rs (12).

### ICKs plasmids construction

Synthetic DNA coding for the light (VL) and heavy chain (VH) variable domains of RTX were chemically synthesized (Geneart GmbH) and cloned into pFUSE2ss-CLIg-hκ and pFUSEss-CHIg-hG1, respectively (*In vivo*Gen). Then, the IL-2no-alpha gene (25) was fused in frame at the 3′ end of the RTX heavy chain (hIgG1) gene. For the irrelevant ICK (anti-MOPC(hγ1)-IL2no-alpha, also termed isotype control ICK) synthetic DNA encoding for the heavy and light chain variable domains of the anti-MOPC-21 antibody (GenBank no. AAD15290.1 and AAA39002.1) (28) were inserted into the same vectors used for RTX ICK (hIgG1) and IL-2no-alpha was similarly fused. Finally, the DNA encoding for the ICKs heavy and light chains were amplified from the previously obtained constructs and independently cloned into pLV-CMV-IRES-Neo, to obtain the lentiviral transfer vectors.

To obtain anti-huCD20(mγ2a)-IL2no-alpha and type I anti-CD20 mouse IgG2a (termed herein anti-CD20), heavy and light chain DNA were assembled *in vitro* by overlapping PCR. In the case of light chain, RTX VL synthetic gene was fused to the mouse Cκ gene. For the murine anti-CD20 antibody, RTX VH synthetic gene was joined to the CH mouse IgG2a gene, while for the ICK, the IL-2no-alpha gene was fused to carboxy terminus of the previously rearranged RTX-mIgG2a heavy chain gene.

The assembled genes were independently cloned into the pLV-CMV-IRES-Neo vector. The recombinant lentiviral transducing particles were used to stably transfect CHO cells. The culture supernatants of the resulting clones were harvested and the recombinant proteins were purified by protein-A chromatography and further size exclusion chromatography. ICK concentrations were determined by measuring the absorbance at 280 nm, and their purity was analyzed by SDS-PAGE and HPLC on a Superdex-200 column.

### SDS-PAGE and western blot analysis

Purified proteins were analyzed on 7-12%, SDS-PAGE in reducing and non-reducing conditions as described previously (29). Proteins were transferred to nitrocellulose membranes (Whatman, USA) by electric field in semi-humid conditions using a Semiphor Transphor Unit (Pharmacia Biotech, USA). For western blot analysis of purified proteins, HRP-conjugated goat anti-human kappa light chain antibodies (Sigma-Aldrich, USA) were used. Goat anti-human IgG (γ chain-specific) (Sigma-Aldrich, USA) or rabbit anti-IL-2 polyclonal (Bio-RAD, EUA) antibodies were used as primary antibodies before HRP-conjugated rabbit anti-goat or goat anti-rabbit antibodies were added as secondary revealing antibodies.

RTX or a murine IgG2a version of RTX (anti-CD20) were used as controls.

### Quantification of plasma ICK and RTX levels

Anti-huCD20(hγ1)-IL2no-alpha and RTX plasma levels were evaluated by a sandwich ELISA. Microtiter plates coated with 3ug/mL of anti-human IgG mAb (Fc specific) (Sigma-Aldrich, USA) were blocked with 4% (w/v) skim milk powder in PBS-T (phosphate buffered saline (PBS); Tween 20 at 0.05%, pH 7.5) (M-PBS-T) for 1 h at 37^0^C. Mouse sera were diluted (1/10 to 1/500) in 0.2% M-PBS-T (from 1/10 to 1/40) and incubated in the plates for 2 h at 37^0^C.

To detect the recombinant proteins, HRP-anti-human IgG (Fab specific) antibodies (Sigma-Aldrich, USA) were used. To quantify the levels of recombinant proteins in plasma, purified ICK or commercial RTX mAb were used as standards (standard curve range: from 0.5 to 37ng/mL). Samples were analyzed in triplicate. After every step of incubation, the plates were washed three times with washing buffer PBS-Tween 0.05%.

### Flow cytometry

For flow cytometry, cell suspensions from spleen were prepared according to standard protocols. All fluorochrome-conjugated mAbs used were from eBioscience, unless otherwise stated; FITC-conjugated anti-CD3 (145-2C11), PECy5.5-conjugated anti-CD4 (L3T4), PE-conjugated anti-NK1.1 (PK136), PE-conjugated anti-CD8 (eBio H35-17.2), PECy5.5-conjugated anti-B220 (RA3-6B2), PE-conjugated anti-Foxp3 (NRRF-30), were from R&D Systems. Intracellular Foxp3 staining sets were purchased from eBioscience. Samples were measured using a Gallios (Becton Dickinson) flow cytometer and analyzed using FlowJo software (TreeStar).

### STAT5 phosphorylation

CTLL-2 cells were incubated for 15 min at 37°C with IL-2-containing molecules. After incubation, cells were immediately fixed with formaldehyde (PanReac AppliChem,Germany) to preserve the phosphorylation status for 10 min at room temperature and permeabilized with methanol (PanReac AppliChem,Germany) for 50 min on ice. The cells were stained with anti-Stat5-p-PE (phycoerythrin, BD Biosciences) antibodies and analyzed by flow cytometry.

### Proliferation assays

CTLL-2 cells (10^4^ cells per well) were seeded in 96-well cell culture plates in RPMI 1640 medium 5 h prior to the proliferation assay. Then, IL-2, IL-2no-alpha mutein or ICKs were added at different concentrations followed by incubation for 48 h. Afterwards, 20μl of alamarBlue™ dye (Bio-Rad) per well were added, and plates were incubated for 12 h. Finally, plates were read at 540 and 630 nm, and the percentage of reduced alamarBlue™ was calculated following the manufacturer’s recommendation.

### Isolation of PBMC

Peripheral blood mononuclear cells (PBMC) from healthy human donors were isolated by density gradient centrifugation using Ficoll-Paque™ PLUS (GE-Healthcare).

### Recognition of CD20 positive cells

EL4-huCD20, EL4, Ramos, Raji and Jurkat cells (2 × 10^5^) and PBMC from refractory low- grade B-cell lymphoma (5× 10^5^) were incubated with ICKs or mAb on ice for 30 min and washed with PBS. The binding of the antibodies was detected by incubation with FITC-conjugated rabbit anti-human IgG F(ab’)_2_ (Dako, Denmark) or with PE-conjugated goat anti-mouse (Fab specific) (Abcam, USA) for 30 min on ice, followed by flow cytometry.

### Complement-dependent cytotoxicity

Cells were incubated with 6nM of ICKs and equimolar amounts of parental molecules (mAbs, IL-2no-alpha) or their combination for 2 h at 37°C in RPMI medium supplemented with 1% bovine serum albumin (BSA). Human AB serum (ABS) from healthy donors was used as the source of complement, and used at 20%. Then, cells were washed, resuspended in PBS with propidium iodide (PI; Sigma-Aldrich) at 10μg/mL and analyzed by flow cytometry. Dead cells were determined by scatter measurement (forward scatter and side scatter) and PI internalization. All cells that gated out of live cells and were PI-stained were considered dead.

### AnnexinV-FITC assay

Annexin V was used to detect early stage apoptosis. Ramos cells (2.5 × 10^4^) were incubated with the ICKs, mAbs, IL-2no-alpha or combination of mAbs and IL-2no-alpha (18nM) for 24 h in RPMI medium supplemented with 2% FBS. Afterwards, cells were washed twice with annexin V-binding buffer and incubated with annexin V-FITC and PI according to manufacturer’s instructions (TACS Annexin V-FITC, TREVIGEN).

### Caspase 3 activation

Ramos cells (3 x 10^4^) were incubated with ICKs (6nM) for 48 h in RPMI medium supplemented with 2% FBS. Cells were washed with PBS and fixed and permeabilized with Cytofix/Cytoperm (BD PharMingen, USA). Then, cells were stained with Alexa Fluor 488-conjugated goat anti-cleaved caspase 3 (Asp175) antibodies (Cell Signaling, USA).

### Antibody-dependent cell-mediated cytotoxicity

Antibody dependent cell-mediated cytotoxicity (ADCC) was measured by an LDH-release assay, as previously described (30). PBMC from a healthy human donor were used as effector cells. Briefly, 2 × 10^4^ Ramos cells were mixed with the effector cells at a 1:10 target:effector ratio in RPMI medium supplemented with 1% FBS. After 4 h incubation with mAbs, combination of mAbs+ IL-2no-alpha or ICKs at 37°C and 5% CO_2_, 100μL of the supernatant were collected. The cytotoxicity detection kit (Roche, Switzerland) was used according to manufacturer’s recommendations. The absorbance of the product was measured at 490 nm with 620 nm filter in an ELISA reader ELX800 (DIALAB GmbH). Maximum release (high control) of LDH was determined in cells treated with 1% Triton X-100, while spontaneous release levels were measured in cells without antibody. Cells incubated with antibodies (low control) and effector cells alone were included as controls. The percentage of specific lysis was calculated according to the following formula:


$$Cytotoxicity\left(\%\right)=\left\{\frac{\left[\left(effector-\;target\;cell\;mix-effector\;cell\;control\right)-low\;control\right]}{high\;control-low\;control}\right\}*100$$

To evaluate the elimination of B cells from a diffuse large B-cell lymphoma (DLBCL) patient, fresh PBMC (3x10^5^ cells) were cultured in presence of 18nM of ICK, RTX or isotype control ICK during 24 h at 37°C and 5% CO2. Then, cells were collected, washed in, stained with anti-CD19-PECy7 (phycoerythrin cyanine-7, BD PharMingen) and analyzed by flow cytometry. As a control, a similar procedure was performed with fresh PBMC from a healthy donor.

### NK cell co-culture in presence of ICK

To study NK cell responses, freshly human PBMC and Ramos cells were cocultured at a 10:1 ratio in presence of 6nM of ICK, RTX or IL-2no-alpha. After a 21-hour incubation period at 37°C and 5% CO_2_, cells were collected, washed in, stained and analyzed by flow cytometry. The following antibodies were used to detect cell surface antigens: anti-CD3-APC (allophycocyanin, BD PharMingen), anti-CD56-FITC (fluorescein isothiocyanate, Becton Dickinson), anti-CD69-PeCy7(phycoerythrin cyanine-7, BD PharMingen), anti-CD16-AF-700 (AlexaFluor 700, Invitrogen) and anti-107a-PE (phycoerythrin, BD PharMingen).

### IFN-γ analysis

IFN-γ concentrations in the supernatants of activation assay were determined using a commercially ELISA kit (MABTECH). A standard calibration curve generated by serial dilutions of recombinant cytokine was used for quantification.

### Immunofluorescence analysis

Ramos cells were mounted on slides (Superfrost Ultra plus Slide, Thermo Scientific) and fixed using 4% paraformaldehyde for 20 min at -20°C. The cells were then incubated with mAbs or ICKs (60nM) for 1 h at 4°C. After incubation for 30 min with FITC-conjugated anti-mouse or anti-human IgG (DAKO), cells were washed in PBS and mounted using the Vectashield^®^ medium with DAPI (Vector Laboratories). Images were collected on a wide-field microscope (Olympus BX51 LTD microscope).

For immunofluorescence detection, the images were analyzed using the ImageJ software (1.43 version). Similar best-fit lower threshold values were determined for each image to reduce signal background, with the upper threshold always set at 255 arbitrary units (A.U). The results are represented by the mean of green fluorescence/10-cells/Image. Representative images were photographed at 100X magnification.

### Mice

Five to eight-week-old female C57Bl/6 mice were obtained from the National Center for Laboratory Animal Breeding (Havana, Cuba). The mice were adapted during 7 days to the environment of the Animal facilities of the Center of Molecular Immunology (CIM) and mice weighing 18–20g were used for the experiments. Food and water were provided *ad libitum*. The experiments were performed according to guidelines of the International Laboratory Animals Resources using standardized procedures in the CIM. All animal studies were conducted under a protocol approved by the Institutional Animal Care and Use Committee and were informed according to The ARRIVE Guidelines 2.0 (https://www.arriveguidelines.org/resources).

### Pharmacokinetics experiments

C57Bl/6 mice were injected intraperitoneally (i.p.) with equimolar doses of anti-huCD20(hγ1)-IL2no-alpha (50 μg) or RTX (41.6 μg). At various time points (up to 312 h), blood samples were taken (3 mice *per* point and *per* treatment) and immediately centrifuged, and the plasma was frozen at –20°C. Proteins plasma levels were evaluated by ELISA (see *Above*) and bioavailability parameters were calculated using a one-compartment model with GraphPad Prism and WinNonlin^®^ version 8.2 softwares.

### EL4-huCD20 tumor model

C57Bl/6 mice were inoculated intravenously (i.v.) in the tail vein with 5 x 10^5^ EL4-huCD20 cells per mouse in 200 µL PBS on day 0 and were randomized using the single method in groups receiving various treatments. Anti-CD20 mAb was administered by i.p injections (200 µg/injection/mouse) at days 1, 4, 7, 10 and 13 andanti-huCD20(mγ2a)-IL2no-alpha ICK (20-50µg) was injected i.p. on days 1, 4, and 7 after tumor inoculation. In some experiments, anti-huCD20(mγ2a)-IL2no-alpha ICK (20μg) and equimolar amounts of anti-CD20 mAb and IL-2no-alpha mutein were injected i.p. on days 1, 4, and 7 after tumor inoculation. In other experiments, RTX and anti-huCD20(hγ1)-IL2no-alpha ICK were injected i.p. on days 1, 4, and 7 after tumor inoculation. Mice were euthanized when signs of disease appeared (eg. prostration, paralysis, body weight drop). In other experiments, RTX and human ICKs were injected after tumor inoculation to evaluate their impact on spleen cells at day 11.

### Statistical analysis

Statistical significance (P< 0.05) was determined by one-way ANOVA or Kruskal-Wallis tests with the Bonferroni or Dunn’s post-test, respectively, for multiple comparisons. To assess survival differences, Kaplan-Meier curves were produced and analyzed by log-rank tests. Statistical analyses were performed with the Prism software (version 7.0, GraphPad) or IBM SPSS Statistics software (version 25, IBM).

## Results

### Design, generation and characterization of anti-CD20-IL2no-alpha immunocytokines

**Figure 1:**
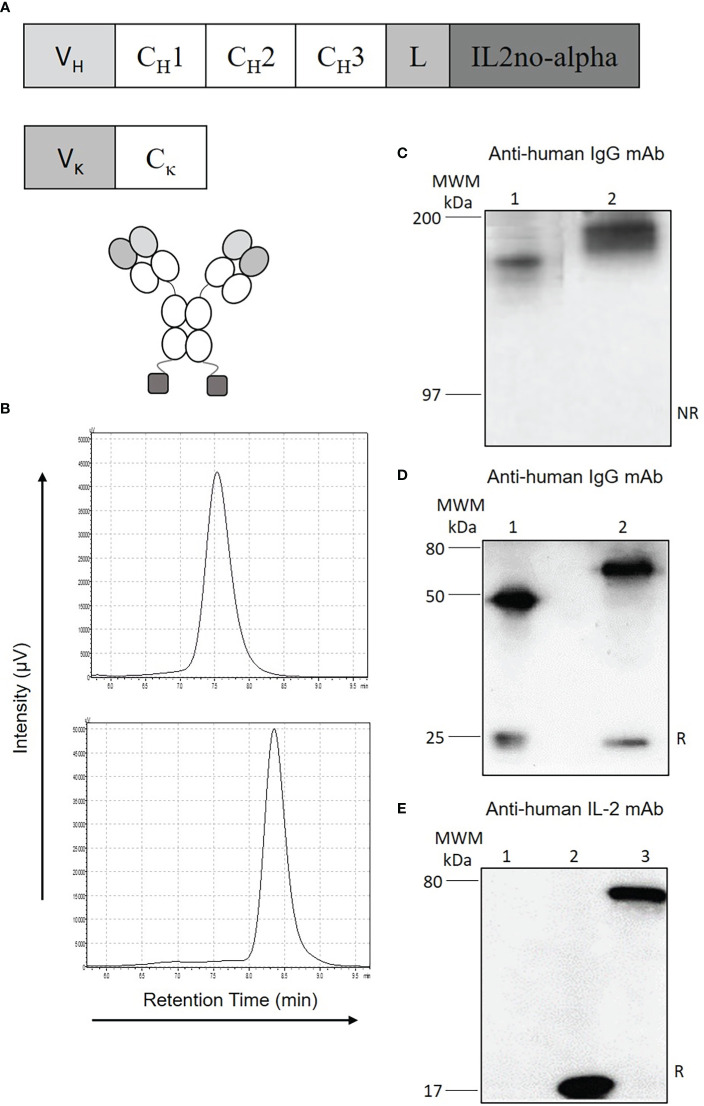
Design and characterization of anti-huCD20(hγ1)-IL2no-alpha ICK. **(A)** Schematic representation of anti-huCD20(hγ1)-IL2no-alpha (CH1, CH2, CH3, constant region of human γ1 heavy chains; L, amino-acid linker (Gly4Ser)_3_GT; V, variable domain of RTX. **(B)** Gel filtration analysis of RTX (upper panel) or purified anti-huCD20(hγ1)-IL2no-alpha (lower panel). **(C–E)**, Western blot analysis using anti-human-IgG antiserum in **(C)** non-reducing conditions and **(D)** reducing conditions (Lane 1: RTX, lane 2: anti-huCD20(hγ1)-IL2no-alpha) or **(E)** using anti-IL-2 rabbit polyclonal antibodies (Lane 1: RTX, lane 2: mutein IL2no-alpha, lane 3: anti-huCD20(hγ1)-IL2no-alpha). MWM, Molecular Weight Marker. NR, non-reducing conditions; R, reducing conditions.

To improve the tumor uptake of the ICKs, a full-length IgG-based format was chosen due to its longer half-life in blood compared to small antibody fusion fragments (31). The ICKs were created to conserve an intact and functional Fc region while keeping bivalent binding of the cytokine to dimeric βγ IL-2R (**Figure 1A**). The size-exclusion chromatography profile also showed a single peak with a retention time corresponding to the apparent molecular mass of the homodimer (**Figure 1B**, lower panel and **Supplementary Figure 1C**). Western-blot analysis confirmed the presence of a single band of apparent molecular weight of 180 kDa in non-reducing conditions (**Figure 1C** and **Supplementary Figure 1A**) and of two bands of 25 and 65 kDa in reducing conditions (**Figure 1D** and **Supplementary Figure 1B**) allowing the identification of anti-huCD20(hγ1)-IL2no-alpha heavy and light chains. The cytokine moiety could be detected with anti-IL-2 rabbit polyclonal antibodies in reducing conditions (**Figure 1E**).

### Anti-huCD20(hγ1)-IL2no-alpha and anti-huCD20(mγ2a)-IL2no-alpha ICKs retain their binding to human CD20

**Figure 2:**
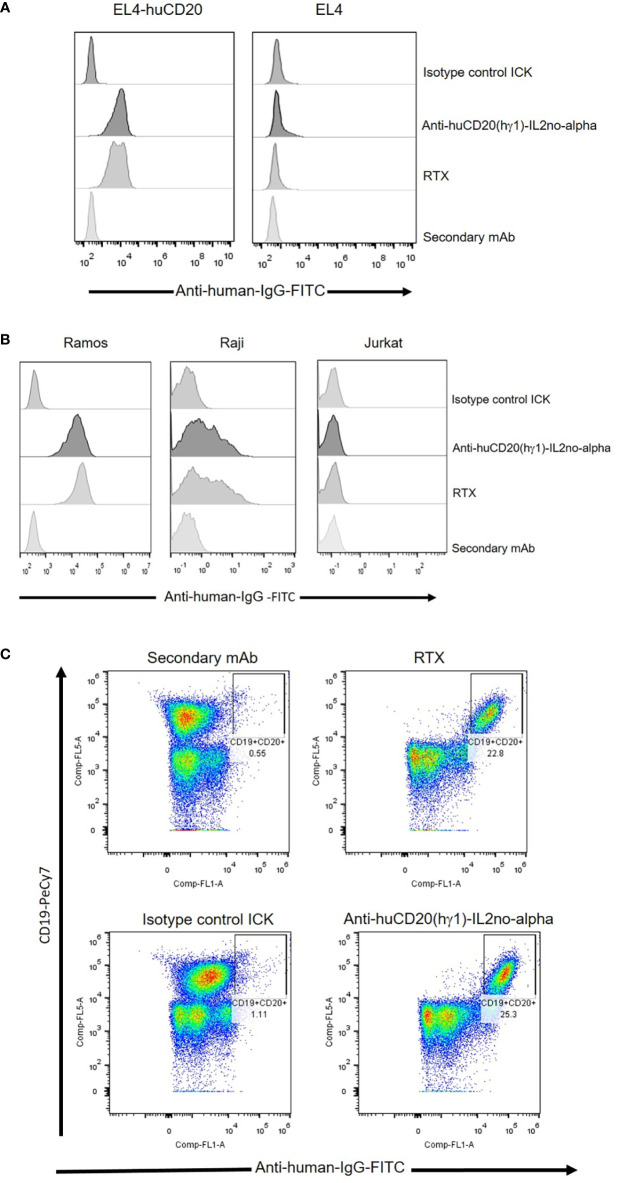
Specific binding of anti-huCD20(hγ1)-IL2no-alpha ICK and RTX antibodies as measured by flow cytometry. **(A)** EL4-huCD20 and EL4 cells. **(B)** Human Burkitt’s lymphoma Ramos and Raji cells were stained with equimolar amounts (66 nM) of anti-huCD20(hγ1)-IL2no-alpha or RTX. Jurkat leukemia cells were used as control of non-CD20-expressing cells. **(C)** PBMC from refractory low- grade B-cell lymphoma. Anti-human IgG-FITC were used as secondary mAb to detect cell-bound antibodies.

Binding of anti-huCD20(hγ1)-IL2no-alpha and anti-huCD20(mγ2a)-IL2no-alpha to CD20 was analyzed by flow cytometry using EL4-huCD20 cells. The CD20 expression levels on these cells are comparable to that of primary human B-NHL or human lymphoma cell lines. ICKs, RTX and anti-CD20-IgG2a antibody bound similarly to EL4-huCD20 cells (**Figure 2A** and **Supplementary Figure 2A**) but not to parental EL4 cells (**Figure 2A** and **Supplementary Figure 2B**).

Also, human Ramos and Raji lymphoma cell lines were used to assess the ability of anti-huCD20(hγ1)-IL2no-alpha to bind to human CD20. As shown in **Figure 2B** and **Supplementary Figure 2C**, the anti-huCD20(hγ1)-IL2no-alpha and RTX share the same recognition profile of CD20-positive cells. Ramos and Raji cells displayed high and low CD20 expression, respectively, as previously reported (32). Jurkat cells and an irrelevant ICK as isotype-matched control were used as negative controls (**Figure 2B**). Next, the binding of anti-huCD20(hγ1)-IL2no-alpha to refractory low-grade circulating B-cell lymphoma cells was evaluated. **Figure 2C** shows that either ICK or RTX exhibited similar percentages of labeled CD20-positive B cells. Irrelevant ICK was used as negative control. Moreover, the presence of the IL-2no-alpha moiety in the anti-huCD20(hγ1)-IL2no-alpha ICK bound to EL4-huCD20 cells was assessed using a FITC-conjugated anti-hIL-2 mAb. The results shown in **Supplementary Figures 3A, B** reveal that the IL-2 specific antibody binds to the cytokine moiety, indicating that fused IL-2no-alpha is exposed and accessible after its binding to the EL4-huCD20 cells.

These observations confirmed that fusion of IL-2no-alpha to anti-CD20 mAbs does not interfere with the binding to CD20.

### Anti-huCD20(hγ1)-IL2no-alpha and anti-huCD20(mγ2a)-IL2no-alpha ICKs contain a functional cytokine

**Figure 3:**
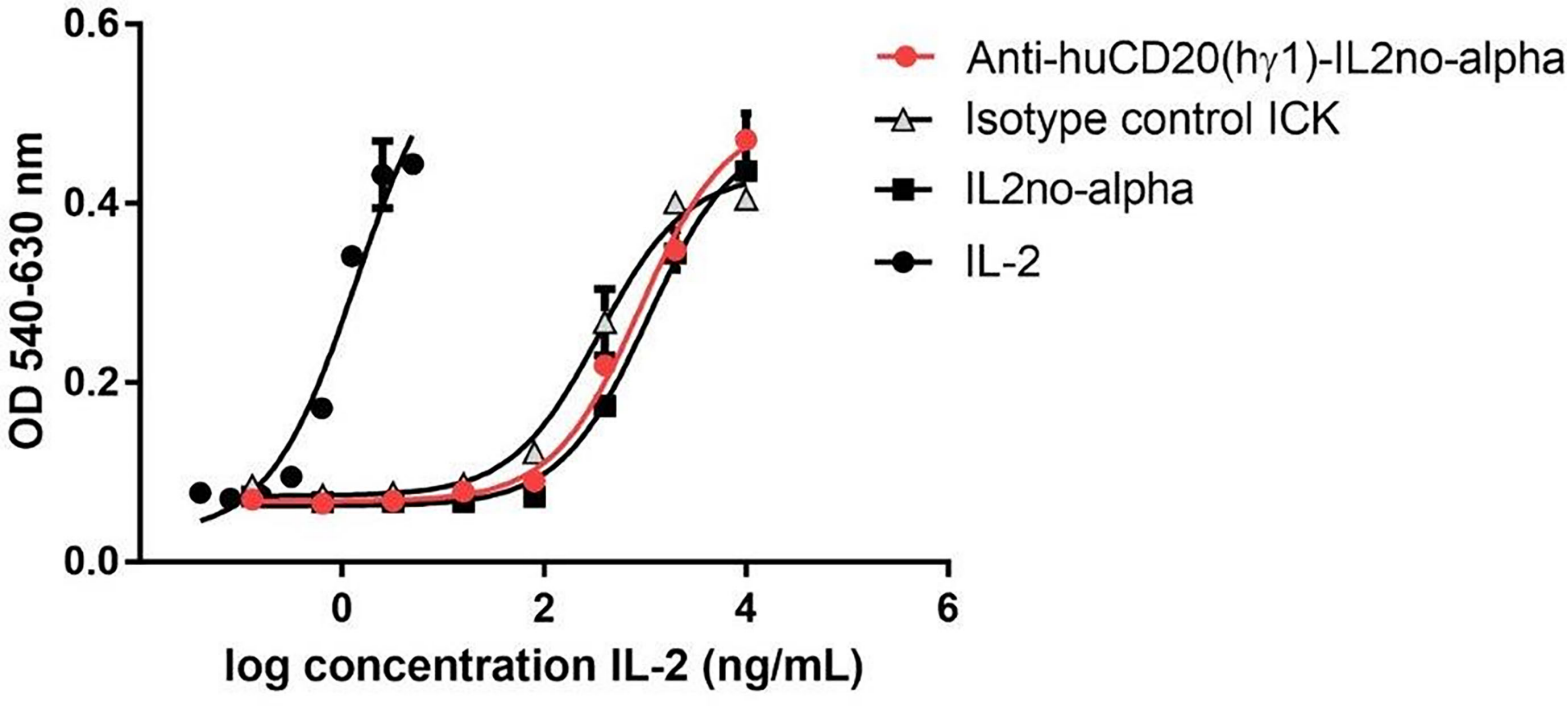
Cytokine-dependent functional effects of anti-huCD20(hγ1)-IL2no-alpha ICK. CTLL-2 cells were also cultured in presence of IL-2no-alpha mutein or with complete medium alone or in presence of IL-2, anti-huCD20(hγ1)-IL2no-alpha, or isotype-control ICK. After 48 h, cell proliferation was assessed by Alamar Blue and expressed as absorbance values (O.D. 540-630nm). Values represent mean ± SEM of cell cultures run in triplicates. At least two independent experiments were performed.

Phosphorylation of STAT5 was then evaluated in CTLL-2 cells, stimulated with equimolar concentrations of cytokines. The IL-2no-alpha mutein and anti-huCD20(mγ2a)-IL2no-alpha were equivalent in their ability to induce phosphorylation of this transcription factor (**Supplementary Figure 4A**). Moreover, the biological activity of these molecules was measured by a CTLL-2 cell proliferation assay. As shown in **Figure 3** and **Supplementary Figure 4B**, respectively, the cell proliferation induced by anti-huCD20(hγ1)-IL2no-alpha and anti-huCD20(mγ2a)-IL2no-alpha was concentration-dependent and comparable to that induced by IL-2no-alpha mutein.

### Antibody effector functions of anti-huCD20(hγ1)-IL2no-alpha ICK

*In vivo* efficacy of ICK therapy might reflect many different mechanisms including the effector functions of the mAb within the fusion protein. Thus, whether the novel ICK represents any advantage over RTX in terms of antibody effector mechanisms was investigated.

**Figure 4:**
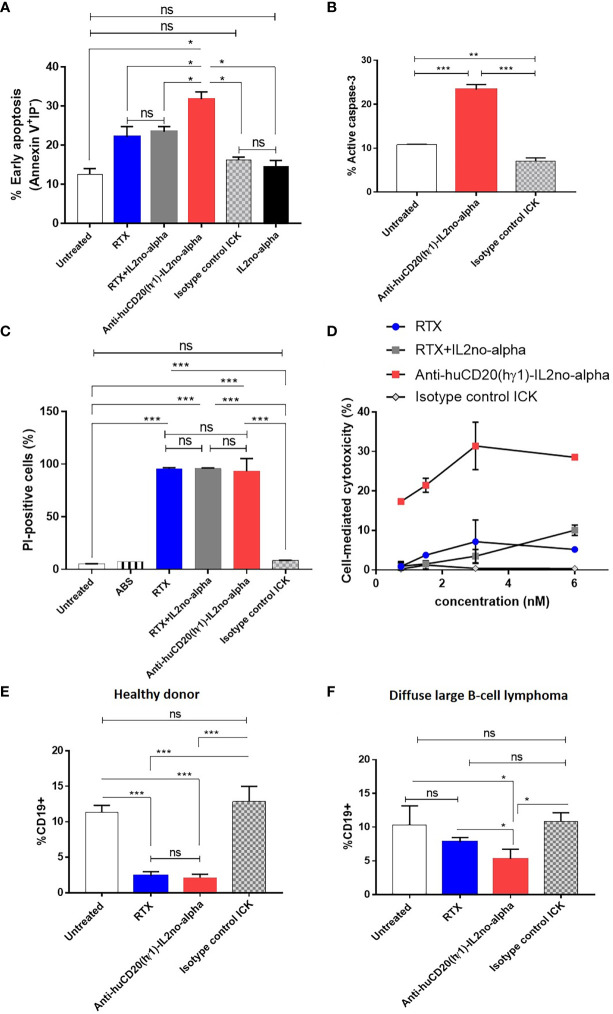
Anti-huCD20(hγ1)-IL2no-alpha ICK is effective in initiating apoptosis, CDC and ADCC. **(A)** Induction of apoptosis by anti-huCD20(hγ1)-IL2no-alpha. Human Burkitt’s lymphoma Ramos cells were incubated with 18nM of mAb or ICKs, or RTX (18nM) + mutein IL-2no-alpha (equimolar amount of cytokine) for 24 h at 37°C. Percentage of early apoptotic cells. **(B)** Late stage apoptosis was measured by activation of caspase 3. Ramos cells were incubated with IL2no alpha based ICKs (18 nM) for 48 h at 37°C. Percentage of cells with active-caspase 3 was determined by intracellular immunofluorescence staining (number of positive cells/number of total cells). MOPC-IL2noalpha was used as an isotype control ICK. Horizontal bars represent the mean ± SEM. **(C)** Induction of complement-dependent cytotoxicity (CDC). Human Burkitt’s lymphoma Ramos cells were treated with 6nM of RTX or ICKs for 2 h at 37°C. Human AB serum was used diluted 1:5. Cell lysis was determined by PI uptake. **(D)** Dose-dependent ADCC against Ramos cells. PBMC from one healthy human donor were used as source of effector cells and cocultured with Ramos cells at a 10:1 ratio, for 4 h at 37°C. Cytotoxicity was measured by an LDH-release assay and expressed in percentage. RTX and isotype control ICK were used as positive and negative controls, respectively. **(E, F**) *In vitro* depletion of B cells from DLBCL patient. PBMC from a healthy donor (**E**, control) and a DLBCL patient **(F)** were incubated in presence of 18nM of ICK, RTX or irrelevant ICK, for 24 h at 37°C. The percentage of B cells within the PBMC was measured by flow cytometry. Data correspond to three independent samples. Data represent the mean ± SEM. (Kruskal-Wallis, Dunn’s *post hoc* test, *P < 0.05; **P < 0.01; ***P < 0.001; ns, not significant).

First, we addressed the capability of inducing apoptosis, a well-described mechanism of action of RTX (1). Then, we evaluated the phosphatidylserine (PS) exposure as it characterizes the early stages of this mechanism (33). Ramos cells were treated with equimolar amounts of anti-huCD20(hγ1)-IL2no-alpha, RTX or RTX plus IL-2no-alpha mutein. After 24 h of culture, cells were stained with annexin V-FITC and PI and analyzed by flow cytometry. Anti-huCD20(hγ1)-IL2no-alpha treatment induced higher percentages of cells expressing phosphatidylserine than RTX at early stage of apoptosis (i.e., annexin V^+^ PI^-^) (**Figure 4A**). Moreover, the targeting of IL-2no-alpha mutein was essential for this enhancement as the combination of RTX and mutein IL-2no-alpha was less effective than the ICK (**Figure 4A**). Because phosphatidylserine externalization can also occur following non-apoptotic events, the cleavage of caspase 3 as a late event in apoptosis was also evaluated. During apoptosis, caspase 3 is processed into two fragments and the 17/19 kDa moiety can be detected by a specific Alexa 488-conjugated antibody. Incubation of Ramos cells with anti-huCD20(hγ1)-IL2no-alpha for 48 h increased the percentage of cells with cleaved caspase 3 (**Figure 4B**). By contrast, there was no difference between untreated cells and isotype control ICK-treated cells that remained unaffected (**Figure 4B**).

Second, the capacity of anti-huCD20(hγ1)-IL2no-alpha to trigger CDC was tested, with human AB serum as a source of complement. The anti-CD20 antibody-cytokine fusion protein was able to mediate CDC to the same extent than RTX or RTX in combination with IL-2no-alpha (**Figure 4C**). This result demonstrates that coupling IL-2no-alpha to Fc region does not hinder the recruitment and activation of complement.

Third, the capacity of anti-huCD20(hγ1)-IL2no-alpha to induce ADCC was evaluated by an LDH assay using human (PBMC) as source of effector cells and Ramos cells as target cells. The ADCC activity of anti-huCD20(hγ1)-IL2no-alpha was dose-dependent and markedly higher than that of RTX or RTX in combination with IL-2no-alpha (**Figure 4D**). Lysis at 6nM was about 30% for anti-huCD20(hγ1)-IL2no-alpha while being about 10% for the other recombinant molecules (**Figure 4D**). Furthermore, this effect was even higher than combination of RTX and IL-2no-alpha pointing out an advantage of cytokine fusing to the antibody. No cytotoxicity was observed with the isotype-matched control mAb or the irrelevant ICK (**Figure 4D**), demonstrating the antigen specificity of the effect. These results point out that the effector-mediating Fc portion of anti-huCD20(hγ1)-IL2no-alpha is functional and displays a biological activity superior to that of RTX.

In another experiment, we studied the *in vitro* capability of depleting malignant cells isolated from one NHL patient by flow cytometry, each being PBMC sample the source of effector and target cells. As shown in **Figure 4E**, both anti-huCD20(hγ1)-IL2no-alpha and RTX depleted normal CD19+ B cells from a healthy donor. Remarkably, only the anti-CD20-ICK significantly reduced the percentage of CD19+ cells among PBMC from a patient with DLBCL **Figure 4F**.

### Engagement and activation of NK cells against CD20^+^ cells by anti-huCD20(hγ1)-IL2no-alpha ICK

**Figure 5:**
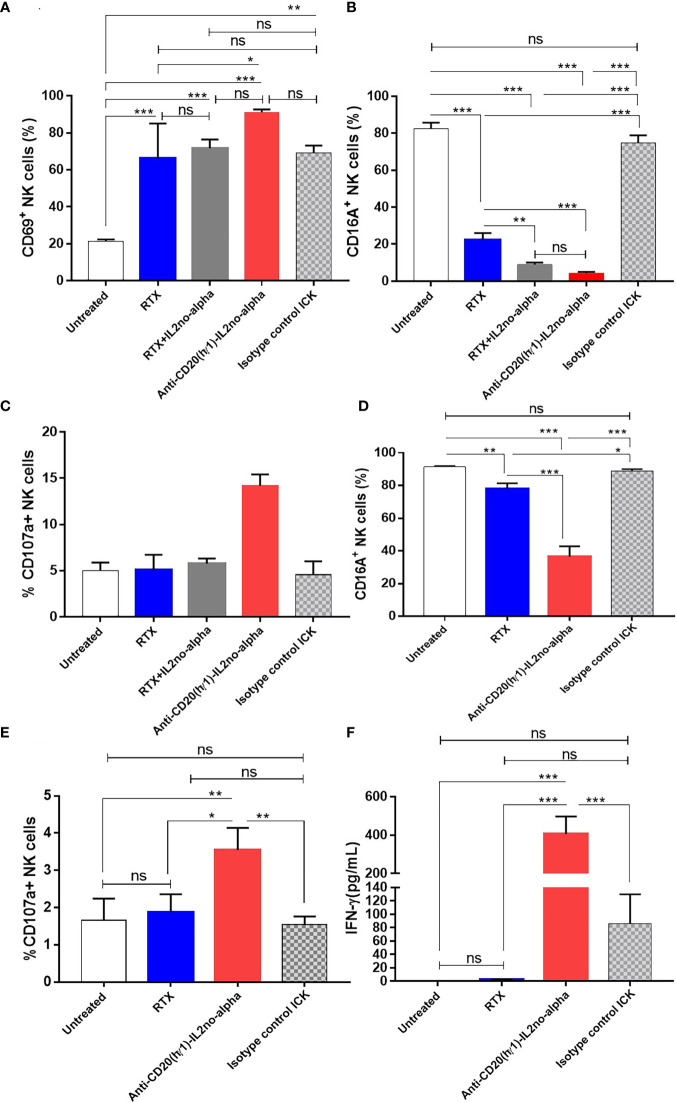
Anti-huCD20(hγ1)-IL2no-alpha ICK induces NK-cell activation and cytotoxic function. **(A–C)**, freshly human PBMC (used as a source of NK cells) and Ramos cells were cocultured at a 10:1 ratio in presence of 6nM ICKs (Anti-huCD20(hγ1)-IL2no-alpha or isotype control ICK), or RTX (6nM), or RTX (6nM) + mutein IL-2no-alpha (equimolar amount of cytokine). Flow cytometry analyses of NK cell (CD3-CD56+) activation membrane markers **(A)**, CD69, **(B)** CD16A, and degranulation marker **(C)** CD107a are shown **(D–F)**, freshly human PBMC and PBMC of refractory low- grade B-cell lymphoma cells were cocultured at a 10:1 ratio for 21 h at 37°C. Flow cytometry analyses of NK cell activation markers **(D)**, CD16A and **(E)** degranulation marker CD107a are shown. Percentage is referred to as the proportion of cells within the whole NK cell population expressing CD69, CD16A or CD107a markers. **(F)** IFN-γ levels in supernatants of cell cultures measured by ELISA. Data represent the mean ± SEM. (Kruskal-Wallis, Dunn’s *post hoc* test, *P < 0.05; **P <0.01; ***P < 0.001; ns, not significant).

Ab-induced NK cell activation is a critical prerequisite for ADCC. Moreover, NK cells have been described as essential mediators of the anti-tumoral effect of IL-2no-alpha mutein (25). Thus, NK cell activation in presence of tumor target cells and of anti-huCD20(hγ1)-IL2no-alpha ICK was assessed. PBMC and Ramos cells were co-cultured at a 10:1 ratio for 20 h in presence of different molecules to evaluate NK cell activation level as measured by surface FcγRIII (CD16) downregulation and CD69 upregulation (34). Similar upregulated expression of CD69 was observed after exposure to RTX, RTX combined with IL-2no-alpha mutein, and to anti-huCD20(hγ1)-IL2no-alpha ICK (**Figure 5A**). In addition, exacerbated downregulation of CD16 occurred after treatment with the ICK or the antibody plus cytokine combination if compared to RTX (**Figure 5B**). The cytotoxic potential of NK cells was then investigated by evaluating the surface expression of lysosome-associated membrane protein-1 (CD107a), a marker of lysosomal degranulation (35). As shown in **Figure 5C**, surface CD107a expression on NK cells was markedly increased only after exposure to ICK. Additionally, PBMCs from a healthy donor and a refractory low-grade B-cell lymphoma patient were co-cultured as above. Similarly, the ICK treatment resulted in significantly downregulated CD16 expression and increased CD107a expression on NK compared with RTX and the isotype control ICK (**Figures 5D, E**). Then, we examined the cell effector response by measuring IFN-γ production. A significant increase of *in vitro* secretion was observed in the presence of anti-huCD20(hγ1)-IL2no-alpha compared with RTX and isotype control ICK (**Figure 5F**). Altogether, these data indicate that convergence of a functional Fc and IL-2no-alpha results in a stronger stimulation of NK cell activity.

### Pharmacokinetics of the anti-huCD20(hγ1)-IL2no-alpha ICK

Before exploring the therapeutic effect of the anti-huCD20(hγ1)-IL2no-alpha ICK in a mouse preclinical tumor model, its pharmacokinetics was studied. Pharmacokinetics of such molecules can be heavily influenced by their format and composition and impact on their antitumor efficacy. In particular, the design of the anti-huCD20(hγ1)-IL2no-alpha ICK involves a mutated form of IL-2 that may reduce its clearance due to its poor interaction with the αβγ IL-2R. Moreover, the presence of the Fc region of the anti-CD20 within the ICK, able to bind FcγRs, could also affect its bioavailability (31).

Thus, female C57Bl/6 mice (n=3) were injected intraperitoneally (i.p.) with a single dose of anti-huCD20(hγ1)-IL2no-alpha (50μg) or RTX (41.6μg) (**Supplementary Figure 5** and **Table 1**). Plasma levels of the anti-huCD20(hγ1)-IL2no-alpha ICK were determined by ELISA. The pharmacokinetics profile of RTX (half-life = 113h) (**Supplementary Figure 5** and **Table 1**) was in agreement with previous reports indicating a half-life of around 100 h in mice (8).

**Table 1:** Summary of the pharmacokinetic parameters of anti-huCD20(hγ1)-IL2no-alpha ICK, RTX and IL-2 in C57Bl/6 mice following a single i.p. administration of a single equimolar dose (0.5 pmol).

Parameter	Anti-huCD20(hγ1)-IL2no-alpha	RTX	IL-2^1^
Mw (kDa)	180	150	15
Dose (μg)	50	41.6	30
Dose (pmol)	0.55	0.55	–
t1/2 (h)	57.7	113.41357	0.42
Tmax (h)	4	8	1
Cmax (μg/ml)	15.305655	20.152173	0.35
AUC (μg/ml*h)	328.45	1361.4732	0.48
Vz/F (μg)/(μg/ml)	5.69	2.9089553	–
CI/F (µg)/(µg/ml)/(h)	0.06795836	0.0177786	–

Mw, molecular weight; Cmax, maximum plasma concentration; Tmax, time to reach maximum plasma concentration; T1/2, half-life; AUC, area under the curve; Vz, volume of distribution; CI, cleareance;^1^Values taken from Bessard et al. (2009) (36).

The values of t_1/2_ (57.7 h), Tmax (4 h) and AUC (328.45 nM.h) of anti-huCD20(hγ1)-IL2no-alpha that half of that of RTX. The volume of distribution (Vd) and clearance (Cl) of ICK are about four-fold lower than RTX. However, both molecules have similar maximum plasma concentration (Cmax) parameters (**Table 1**). In any case, the half-life time of ICK is markedly different to that of mutein IL-2no-alpha (unpublished data) and IL-2 (0.42h) (36), as expected. This variable was previously found to be similar for both the mutein and wild type IL-2 (manuscript in preparation). The pharmacokinetic parameters of anti-huCD20(hγ1)-IL2no-alpha are far higher than those previously measured for IL-2 and IL-2no-alpha cytokines. These results indicate that the fusion procedure significantly changes the bioavailability of the cytokine (**Table 1**).

### Anti-tumor activity of the ICK treatment

**Figure 6:**
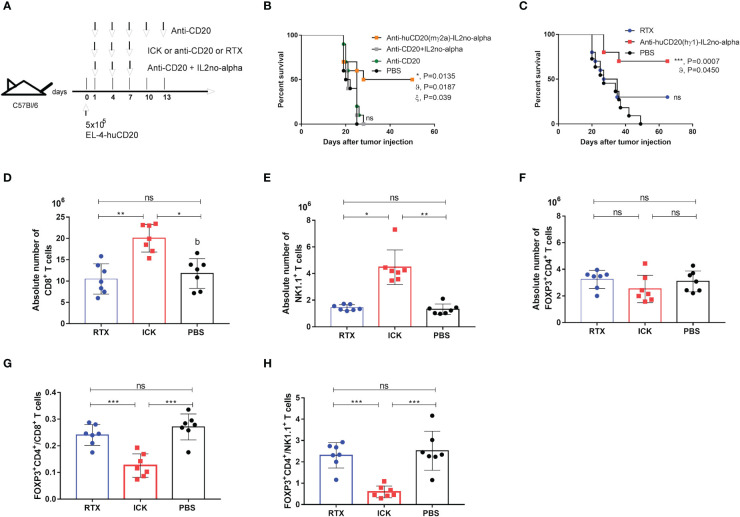
Effect of anti-huCD20(mγ2a)-IL2no-alpha and anti-huCD20(hγ1)-IL2no-alpha ICKs on survival of EL4-huCD20 tumor-bearing C57Bl/6 mice. **(A)** Schematic representation of treatment schedule. **(B)** Survival curves of mice with the different treatments as indicated: saline, anti-CD20(16.6µg), anti-CD20 (16.6µg) + mutein IL-2no-alpha (3.3µg), anti-huCD20(mγ2a)-IL2no-alpha (20 µg). Data correspond to a representative experiment of two independent experiments (n=10 per group). (log-rank test; *, P< 0.05, anti-huCD20(mγ2a)-IL2no-alpha vs PBS; ϑ, P< 0.05, anti-huCD20(mγ2a)-IL2no-alpha vs (anti-CD20 + mutein IL-2no-alpha); ξ, P< 0.05, anti-huCD20(mγ2a)-IL2no-alpha vs anti-CD20; ns: not significant). **(C)** Low doses of anti-huCD20(hγ1)-IL2no-alpha (20ug) are more potent than equimolar dose of RTX (16µg). Data correspond to a representative experiment of two independent experiments (n=10 per group). (log-rank test; ***, P< 0.001, Anti-huCD20(hγ1)-IL2no-alpha vs PBS; ϑ, P< 0.05, Anti-huCD20(hγ1)-IL2no-alpha vs RTX; ns: not significant)). **(D–H)** Anti-huCD20(hγ1)-IL2no-alpha treatments expands NK+ and CD8+ cells over Tregs+. Absolute number of CD8+ T **(D)** NK1.1+CD3+ **(E)** and FOXP3+CD4+ **(F)** cells of splenocytes from C57Bl/6 mice evaluated by flow cytometry 11 days after challenge with 5x10^5^ EL4-huCD20cells and treated with different therapies. **(G, H)** Mice receiving anti-huCD20(hγ1)-IL2no-alpha ICK showed lower ratio of FOXP3+ CD4+/CD8+ T **(G)** and FOXP3+CD4+/NK1.1+ CD3- **(H)** cells. Data correspond to a representative experiment of two independent experiments. Each symbol represents an individual mouse. Horizontal bars represent the mean ± SEM. (Kruskal-Wallis, Dunn’s *post hoc* test *P < 0.05; **P < 0.01; ***P < 0.001; ns, not significant).

We previously reported that anti-CD20 (mIgG2a) in combination with high doses of mutein IL-2no-alpha significantly increases the survival of immunocompetent C57Bl/6 mice challenged with EL4-huCD20 cells as compared to animals treated with the antibody alone or in combination with IL-2. Also, we have shown that no-alpha mutein alone does not increase the survival of the animals in this setting (27). Thus, we evaluated the antitumor effect of the anti-huCD20(mγ2a)-IL2no-alpha in the same tumor model (**Figure 6A**). Initial experiments demonstrated that anti-huCD20(mγ2a)-IL2no-alpha given at days 1, 4 and 7 after tumor inoculation achieved a protection of the animals as efficiently as higher doses of the parental mAb (200µg, at days 1, 4, 7, 10 and 13) (**Supplementary Figure 6A**). When the tumor-bearing mice were treated with three low doses (20µg/injection) of anti-huCD20(mγ2a)-IL2no-alpha, only animals of the ICK-treated group survived in contrast to animals treated with PBS, parental mAb (16.6µg/injection) or a combination of anti-CD20 and IL-2no-alpha given at equivalent molar doses (**Figure 6B** and **Supplementary Table 1**). Low doses of parental mAb or combination of equimolar amounts of mAb and mutein IL2no-alpha failed to prolong survival of any animals (**Figure 6B** and **Supplementary Table 1**).

Next, we evaluated the *in vivo* efficacy of the human version of the anti-CD20 ICK in the same mouse tumor model since the Fc region of human IgG1 can efficiently engage mouse FcγRs (37). Immunocompetent C57Bl/6 mice were i.v. injected with 5x10^5^ EL4-huCD20 cells on day 0, followed by i.p. injection on days 1, 4, 7 of anti-CD20 ICK (20µg/injection) or RTX (150μg/injection) (**Supplementary Figure 6B**). This dose of mAb corresponds to the standard dose of 375 mg/m^2^ used in the clinic (38). A similar survival rate was observed between mice treated with high doses of RTX and mice that received low doses of anti-huCD20(hγ1)-IL2no-alpha ICK (20µg/injection) (**Supplementary Figure 6B**). In contrast, 16.6µg of RTX had no antitumor effect as opposed to the injection of equimolar amount of anti-huCD20(hγ1)-IL2no-alpha ICK (20µg/injection) (**Figure 6C** and **Supplementary Table 2**).

Thus, all these data confirm the greater efficacy of the anti-CD20-IL2no-alpha mutein formats over the parental molecules and their combination.

### Anti-huCD20(hγ1)-IL2no-alpha triggers the expansion of CD8^+^ T cells and NK cells but not of regulatory T cells

To investigate the immune mechanisms underlying the increased survival of tumor-bearing mice treated with anti-huCD20(hγ1)-IL2no-alpha we studied several immune cell populations in the same tumoral setting. After challenging the animals with EL4-huCD20 cells and injecting anti-huCD20(hγ1)-IL2no-alpha, RTX or PBS, mice were sacrificed at day 11 and spleens were collected for further analysis by flow cytometry. The number of total splenic CD8^+^ T cells (**Figure 6D**) and NK cells (**Figure 6E**) was significantly increased in anti-huCD20(hγ1)-IL2no-alpha-treated groups unlike the RTX and PBS groups. In contrast, no difference was found between the number of Tregs (FoxP3^+^ CD4^+^) (**Figure 6F**). It is noteworthy that lower ratios of FoxP3^+^CD4^+^/CD8^+^ and FoxP3^+^CD4^+^/NK1.1 cells were observed in animals receiving anti-huCD20(hγ1)-IL2no-alpha as compared to those injected with RTX or PBS (**Figures 6G, H**). These findings evidence for the first time that anti-huCD20(hγ1)-IL2no-alpha shifts the balance towards effector immune cells rather than Tregs, in line with the results obtained upon the combination of anti-CD20 with higher doses of IL-2no-alpha mutein (27).

## Discussion

Although efficacy and success of RTX has revolutionized B-NHL treatment, many lymphomas fail to respond or eventually show signs of resistance to the therapy (2). We report herein for the first time the development of an IL-2 mutein (no-alpha)-based tri-functional immunocytokine targeting CD20 and engaging FcγRs. This molecule could be used as a new therapeutic tool to improve outcome or overcome refractoriness to antibody therapy.

The antitumoral activity of the ICK at doses where the parental molecules or their combination had no protective effect (**Figure 6B**) supports the rationale of proposing the use of the human IgG1 format anti-CD20 ICK variant for a clinical scenario. The availability of anti-CD20-IL2no-alpha (mouse IgG2a) enabled us to prove the superiority of the novel fusion protein in a context of immune competence, without the interference of mouse antibody responses to human ICKs. Antitumor effects have already been explored for an anti-CD20 IL-2-based ICK but only in a SCID mouse model (7) and included a wild-type version of IL-2, which elicits adverse events similar to those of IL-2 (14). Indeed, DI-Leu16-IL-2, when given subcutaneously in a Phase 2 clinical trial, showed adverse events and an expansion of Tregs that could be detrimental to the anti-tumor activity of the drug (16).

These findings suggest that the fusion to anti-CD20 antibodies of the mutein IL-2no-alpha that expands preferentially CD8^+^ T lymphocytes and NK cells over Tregs with less toxicity than IL-2 (25), may be a good effective therapeutic against human lymphomas. The generation of ICKs bearing IL-2 variants to expand preferentially immune effector cells and to reduce toxic side effects of IL-2 based ICKs has already been explored (4, 14, 23, 24). A CEA-IL2v with diminished α IL-2R binding, and thus preferential β IL-2R association that is predominant on cytotoxic T cells, and a silent Fc region was developed to reduce Tregs response and unspecific activation of immune cells. CEA-IL2v strongly expanded NK cells and CD8^+^ T cells while Tregs proliferation was greatly reduced (14). The same group also fused this IL-2v to PD1- and FAP (fibroblast activation protein α) - targeting antibodies to direct IL-2 delivery to effector cells (4, 23). They showed that PD1-IL2v induced a strong potentiation of T cell response and anti-tumor efficacy as compared to the combination of PD-1 checkpoint inhibition with a non-PD-1-targeted ICK. On the other hand, FAP-IL2v was less potent than FAP-IL2wt in activating immunosuppressive Tregs while it behaves as an effective partner for combination with other immunotherapies. However, CEA-IL2v and FAP-IL2v clinical developments have been stopped due to the lack of clear therapeutic benefit (22). These adverse outcomes do not deny the suitability of IL-2no-alpha targeting, but suggest the need of exploring new formats or combination approaches to face more challenging scenarios like solid tumors.

These latter antibody-cytokine fusion proteins exhibit certain design features oriented to improve the pharmacokinetic properties, toxicity profile and therapeutic effect. They consider neither the bivalence of IL-2 mutants (only one variant of IL-2 molecule per ICK) in the structure generated nor the recruitment of the effector functions of the antibody (4, 14, 23, 24, 39). In contrast, the ICK presented herein (to be used against certain hematological malignancies) adopts a tri-functional format involving effects through direct binding of the antigen CD20, receptor-engagement of the mutated cytokine and cellular functions initiated *via* Fc region within the ICK. Coupling two IL-2 molecules has the advantage of augmented immune modulation compared to ICKs with only one IL-2 molecule (40). To the aforementioned advantages it can be added the extension of the circulatory half-life, for the cytokine, as a consequence of FcRn-mediated antibody recycling (41). In concordance with that, our results pointed out a longer half-life of the immunocytokine, respect to the mutein alone, while lower when compared to RTX (**Table 1**), which has been previously reported for other Ab-cytokine fusion proteins with IgG full-length format (7, 10).

The format of ICK has a significant impact on its targeting activity. First, we showed that fusing IL-2no-alpha did not alter the recognition of CD20 by the antibody portion. The dual variable regions of intact IgG grants ICK a high avidity for its specifc target, which permits for high rates of retention at the site of interest (42) (**Figure 2**). In addition, as for other ICK with similar formats (7, 9), fusion of mutein IL2no-alpha to the carboxy-terminus of the heavy chain does not impede its proliferative activity, demonstrating the compatibility of the design with binding and cytokine moiety activity (**Figure 3**).

Direct induction of apoptosis and CDC have been shown to contribute to the antitumor activities of RTX (1). Surprisingly, we found that *in vitro* anti-huCD20(hγ1)-IL2no-alpha treatment of lymphoma cells induced higher levels of early apoptosis than RTX (**Figure 4A**). Moreover, previous experiments in our lab, in similar conditions (43), have shown that RTX used at the same concentration than in our experiment did not induce the cleavage of caspase 3, which also indicates a potential superiority of the ICK compared to RTX. There is no clear explanation yet for this effect. It was previously reported a very low expression of intermediate affinity IL-2R by normal B cells (44) and Ramos cells (45). Although isotype control ICK did not bind Ramos or refractory low-grade circulating B-cell lymphoma cells (**Figures 2B** and **C**), we cannot exclude that the anti-CD20 ICK could trigger a weak signaling in B cells expressing low density of βγ IL-2R receptor. Hence, further experiments need to be performed to evaluate whether the anti-huCD20(hγ1)-IL2no-alpha ICK can trigger B cell activation.

Interestingly, the complement-dependent cytotoxicity of the anti-huCD20(hγ1)-IL2no-alpha was maintained (**Figure 4C**), as opposed to other IL-2-based ICKs including the anti-CD20-IL2 (Di-Leu16-IL-2) (7) that exhibited a reduced CDC activity as a consequence of coupling IL-2 to the C- terminus of the heavy chain (7, 31).

In particular, IL-2no-alpha mutein has been reported to activate NK cells (25) that exert ADCC *via* the engagement of FcγRIIIa (CD16a) which starts a sequence of events ending in the secretion of IFNγ and granzyme-containing granules (46). In agreement with that, we found that anti-huCD20(hγ1)-IL2no-alpha shows even greater ADCC than RTX alone *in vitro* (**Figure 4D**). This enhanced ADCC is remarkably important as this mechanism is the dominant determinant of efficacy of mAbs in NHLs (47, 48). CD16 downmodulation on NK cells has been observed after RTX-induced ADCC supporting the internalization of the FcγR following Fc-FcR binding (49). Furthermore, it has been reported that loss of CD16 from NK cell surface can allow NK cells to detach from their targets to enhance serial killing (50). As it was expected, anti-huCD20(hγ1)-IL2no-alpha increased NK cell activation upon co-incubation with human Burkitt’s lymphoma cells, as shown by the upregulation of CD69, the downregulation of CD16 and increased levels of CD107a (**Figure 5**). Moreover, direct action of ICK was responsible for similar effects in NK cell population as well as the production of IFN-γ by immune effectors in presence of refractory low-grade B cell lymphoma cells (**Figure 5**). This evidence suggests potential benefits in the response to therapy, in concordance with the improved anti-tumor efficacy in mice after combining immunotherapeutic approaches to activate NK cells and enhance ADCC (10). Furthermore, this enhanced NK cells activation is in agreement with the ability of anti-huCD20(hγ1)-IL2no-alpha to kill B cells from a DLBCL patient, as opposed to RTX (**Figure 4E**). It suggests that the activation of NK cells by the IL2no-alpha moiety of the anti-huCD20(hγ1)-IL2no-alpha could circumvent the limitations encountered when RTX is used such as FcγRIII (CD16) genetic polymorphism, low CD16 expression, inhibitory KIR/HLA interactions, among others (51). This potential advantage of the fusion protein over the Mab, needs to be further confirmed in a larger set of samples from NHL patients.

With regard to our *in vivo* studies, immunocompetent mice were used thanks to the availability of the EL4-huCD20 (27, 38, 52, 53). These tumor cells have shown no intrinsic immunogenicity when injected i.v in C57Bl/6 mice (52, 53). This setting allows the recruitment of a full range of host innate and adaptive immune effector mechanisms. We demonstrate herein that low dose of anti-huCD20(hγ1)-IL2no-alpha, unlike RTX, significantly augments the overall survival of mice challenged with EL4-huCD20 cells as compared to animals treated with PBS (**Figure 6**). This therapeutic effect could be related, in addition to antibody effector functions, to the immunomodulation induced by the ICK characterized by the increase of NK and CD8^+^ T cells but not Tregs (**Figures 6D–H**). We also previously showed (27) that a mouse IgG2a version of RTX at high doses induces a preferential expansion of effector cells over Tregs only at day 21, an effect that is not observed at day 14. In our present experiments, we observed an increase of the effector cells (NK or T CD8+)/Treg ratio provoked by anti-CD20 ICK at day 11. Then, future experiments could be designed to evaluate whether high doses of RTX reproduce the immunomodulatory effect of the ICK and whether it follows the same kinetics remain to be performed.

Some clinical studies have reported that tumor-specific T cell responses could also be detected after the use of RTX in clinical treatment (54, 55). In addition, preclinical studies have shown that the anti-tumor long term effect of anti-CD20 treatment is both CD4^+^ and CD8^+^ T cell-dependent and requires the presence of NK cells (52, 53, 56), whose expansion correlated with better clinical outcome to RTX treatment (13, 57). Moreover, a preclinical study has shown that splenic CD4^+^FoxP3^+^ Tregs expanded in untreated mice that exhibited a reduced survival, whereas Tregs depletion led to long-term survival of the animals (53). The manipulation of Tregs has become indeed the focus of attention as they are significantly increased in peripheral blood of NHL patients receiving or not chemotherapy (58) and have been associated with poor prognosis (19, 21, 52). In an *in vivo* experimental setting similar to the one used here, it has been demonstrated that anti-CD20/no-alpha mutein combination improves the survival rate of CD20^+^ tumor-bearing mice achieved by anti-CD20 treatment with an enhanced cytotoxic potential of NK cells and CD8^+^ T lymphocytes (27).

In summary, our work proposes a new potential novel drug that may offer several advantages over others included in the arsenal of anti-CD20 therapies. While most of next-generation anti-CD20 antibodies exhibit potentiated ADCC, apoptosis or CDC (1), this anti-CD20 ICK displays an enhancement of two of the major mechanisms reported for RTX: ADCC and apoptosis. Thus, it is rather similar to obinutuzumab (59), which is currently used in the first line therapy of patients with Chronic Lymphocytic Leukemia and in the first and second line therapy of patients with Follicular Lymphoma (3, 59). However, unlike this mAb, in addition to upgraded effector functions, this new molecule provides the immunostimulatory effect of no-alpha mutein whose design allows a preferential direct action on NK and effector T cells. RTX resistance frequently occurs in B-cell NHL treated patients. This phenomenon is due, among other reasons, to partial or total loss of CD20 and can be a consequence of anti-CD20 mAb therapy (59). Hence, taking into account that no-alpha mutein in the context of ICK efficiently activates NK cells, this could favor NK mediated ADCC, reverting RTX resistance in patients with problems with ADCC due to above-mentioned causes.

To our knowledge, this is the first study describing fusion of IL-2no-alpha to an anti-CD20 mAb and outlining the therapeutic benefit in the clinic that might increase the number of good responder patients in the first line, administered as combination with chemotherapy. In addition, anti-huCD20(hγ1)-IL2no-alpha would also be expected to be suitable for patients whose resistance to RTX therapy is known to be related to a failure of or non-optimal apoptosis and ADCC.

## Data availability statement

The raw data supporting the conclusions of this article will be made available by the authors, without undue reservation.

## Ethics statement

The studies involving human participants were reviewed and approved by Ethical Comitted of Hermanos Ameijeiras Hospital. The patients/participants provided their written informed consent to participate in this study. The animal study was reviewed and approved by Institutional Animal Care and Use Committees of the CIM.

## Author contributions

AC, KL and TH contributed to conception or design of the work. AC, BC, WD, MG, TG, BF, AG, NL, KS, KC, AL, CP, YR, J-LT, KL and TH contributed to acquisition, analysis or interpretation of the data. AC, J-LT, KL and TH have drafted the work and all authors have revised the manuscript. All authors contributed to the article and approved the submitted version.
