# Supplementary material for: Potent immunomodulatory and antitumor effect of anti-CD20-IL2no-alpha tri-functional immunocytokine for cancer therapy
Source: Front Immunol. 2022 Dec 9;13:1021828. doi: 10.3389/fimmu.2022.1021828 (PMC9780377; doi:10.3389/fimmu.2022.1021828)
Supplement: Supplementary file 1 [file DataSheet_1.pdf]

Supplementary Material

Supplementary Figures

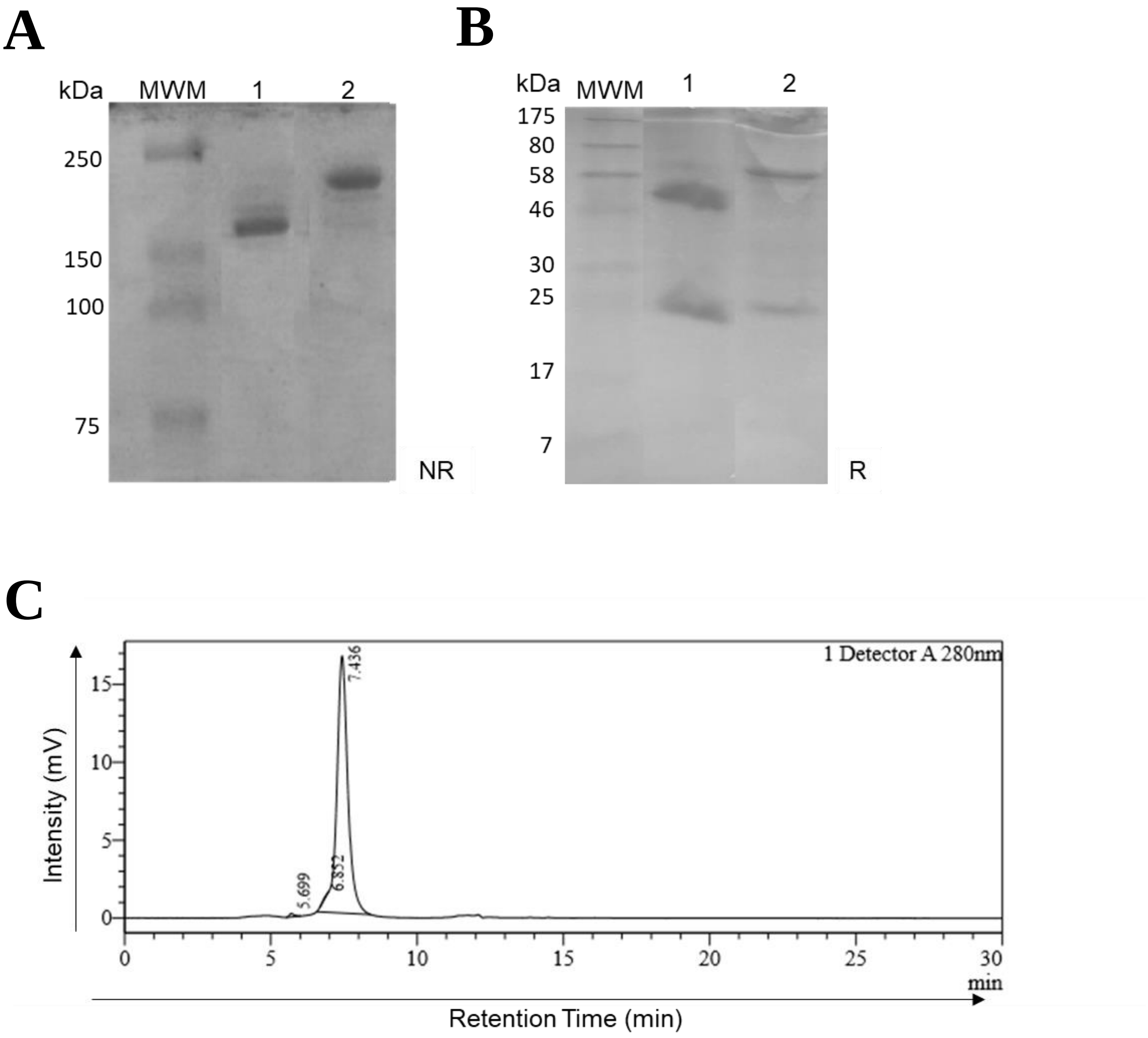

Supplementary Figure 1. Characterization of anti-huCD20(mγ2a)-IL2no-alpha ICK. **(A-B)** SDS-PAGE under **(A)** non-reducing conditions and **(B)** reducing conditions (Lane 1: 5µg anti-CD20, lane 2: 5µg anti-huCD20(mγ2a)-IL2no-alpha ICK). **(C)** Gel filtration analysis of purified anti-huCD20(mγ2a)-IL2no-alpha ICK. MWM: Molecular Weight Marker. NR: non-reducing conditions; R: reducing conditions.

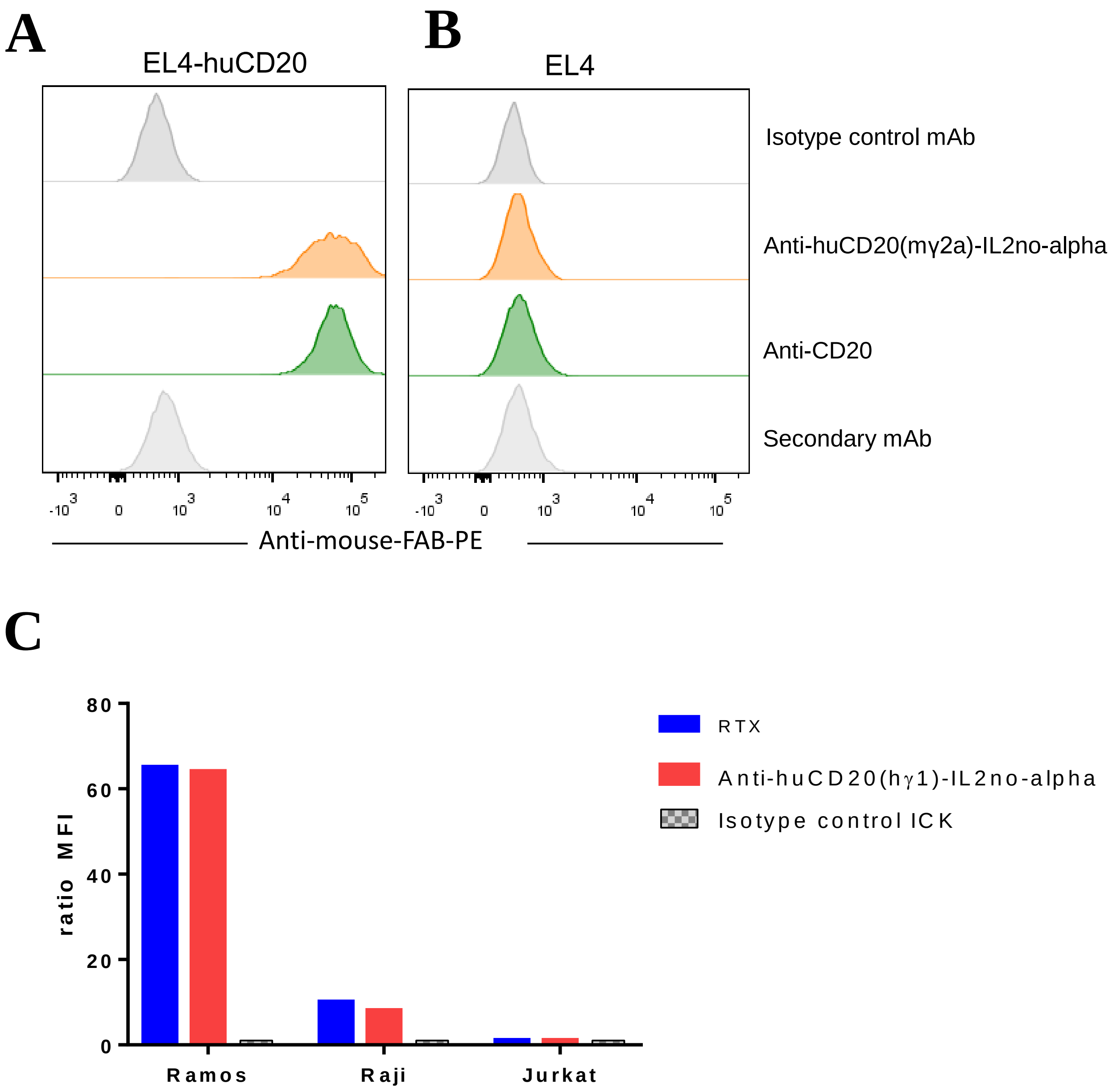

Supplementary Figure 2. Specific binding of anti-huCD20(mγ2a)-IL2no-alpha ICK and anti-CD20 antibody revealed by immunofluorescence flow cytometry. **(A)** Binding to EL4-huCD20 and **(B) to** EL4 cells. **(C)** Ratio of mean fluorescence intensity (MFI) for RTX, anti-huCD20(hγ1)-IL2no-alpha ICK and isotype control ICK *versus* the MFI of secondary mAb when cells from human Burkitt's lymphoma Ramos and Raji cell lines were tested in indirect immunofluorescence assays. Jurkat leukemia T cells were used as control of non-CD20-expressing cells.

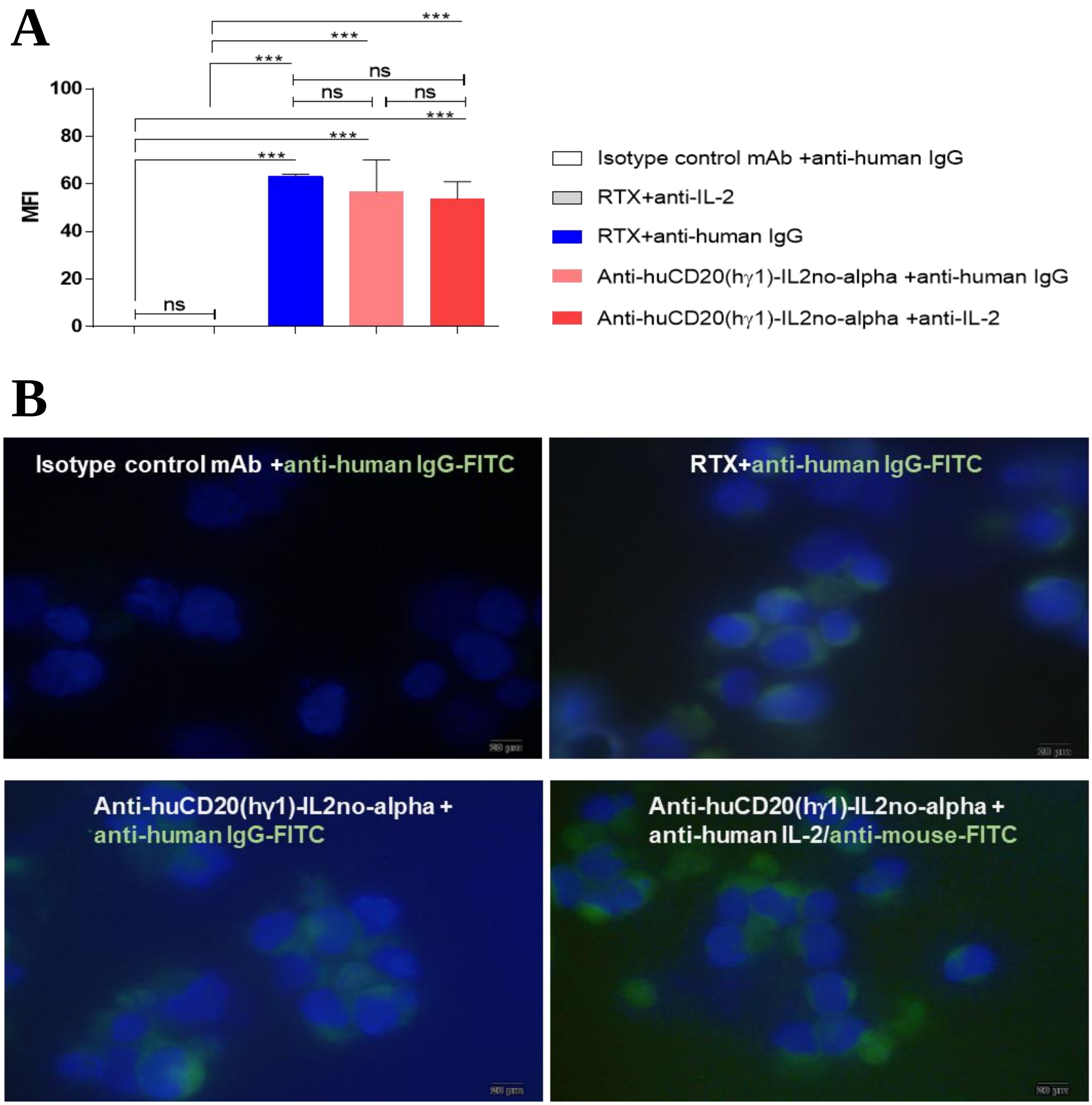

Supplementary Figure 3. Accessibility of the mutein IL-2no-alpha present in the anti-huCD20(hγ1)-IL2no-alpha ICK, as measured by immunofluorescence microscopy. EL4-huCD20 cells were labelled with RTX, or an isotype-matched irrelevant IgG1 antibody or with the anti-huCD20(hγ1)-IL2no-alpha. The binding of the ICK to CD20 was detected with either a mouse anti-human IL-2 mAb and revealed with anti-mouse IgG-FITC antibodies, or directly with a goat anti-human IgG-FITC. The binding of RTX and isotype-matched irrelevant IgG1 antibody was revealed with the goat anti-human IgG-FITC antibody. **(A)** Quantitative evaluation of the binding by immunofluorescence staining. Data are represented as mean±SEM. (One-way ANOVA, Bonferroni post hoc test; ***, $P < 0.001$; ns: not significant). **(B)** Representative photomicrographs of anti-huCD20(hγ1)-IL2no-alpha or RTX positive cells at 100X magnification.

A

B

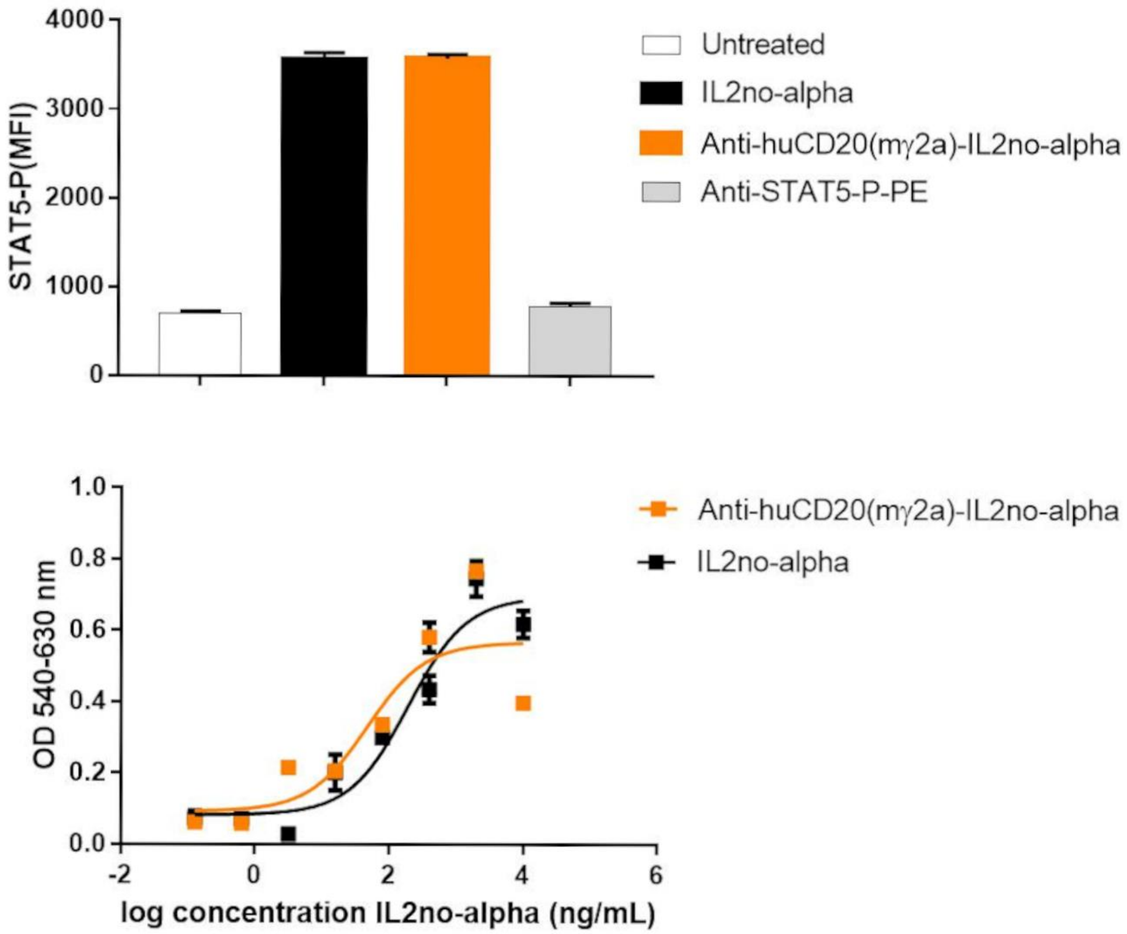

Supplementary Figure 4. Cytokine-dependent functional effects of anti-huCD20(mγ2a)-IL2no-alpha ICK. **(A)** Phosphorylation of STAT-5 in CTLL-2 cells stimulated during 30 min with IL2no-alpha or anti-huCD20(mγ2a)-IL2no-alpha was evaluated by immunofluorescence flow cytometry. Baseline levels of phosphorylation (MFI) were detected with untreated cells labeled or not with an anti-STAT-5-P-PE mAb. **(B)** CTLL2 cells were cultured with complete medium alone or stimulated with graded concentrations in presence of IL-2, mutein IL2no-alpha and anti-huCD20(mγ2a)-IL2no-alpha. After 48h, cell proliferation was assessed by Alamar Blue and expressed as absorbance values (O.D. 540-630 nm). Values represent mean±SEM of cell cultures run in triplicates. Findings were confirmed in at least two independent experiments.

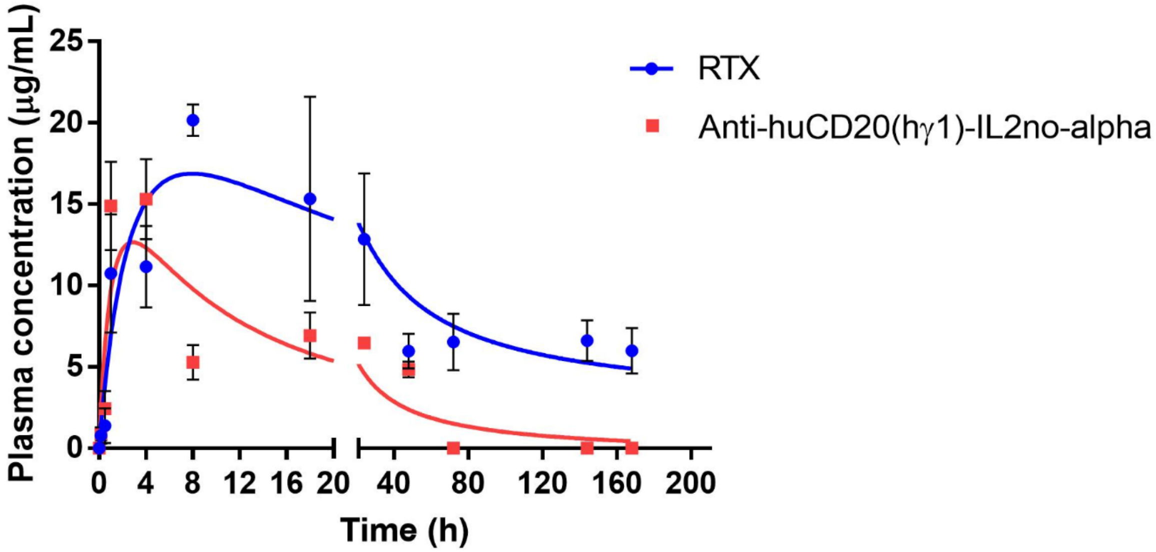

Supplementary Figure 5. Pharmacokinetic profiles of anti-huCD20(hγ1)-IL2no-alpha ICK. Male C57Bl/6 mice were injected with a single intraperitoneal injection of anti-huCD20(hγ1)-IL2no-alpha ICK (50µg) or RTX (46.2µg). Plasma concentrations were determined by ELISA at the indicated time points.

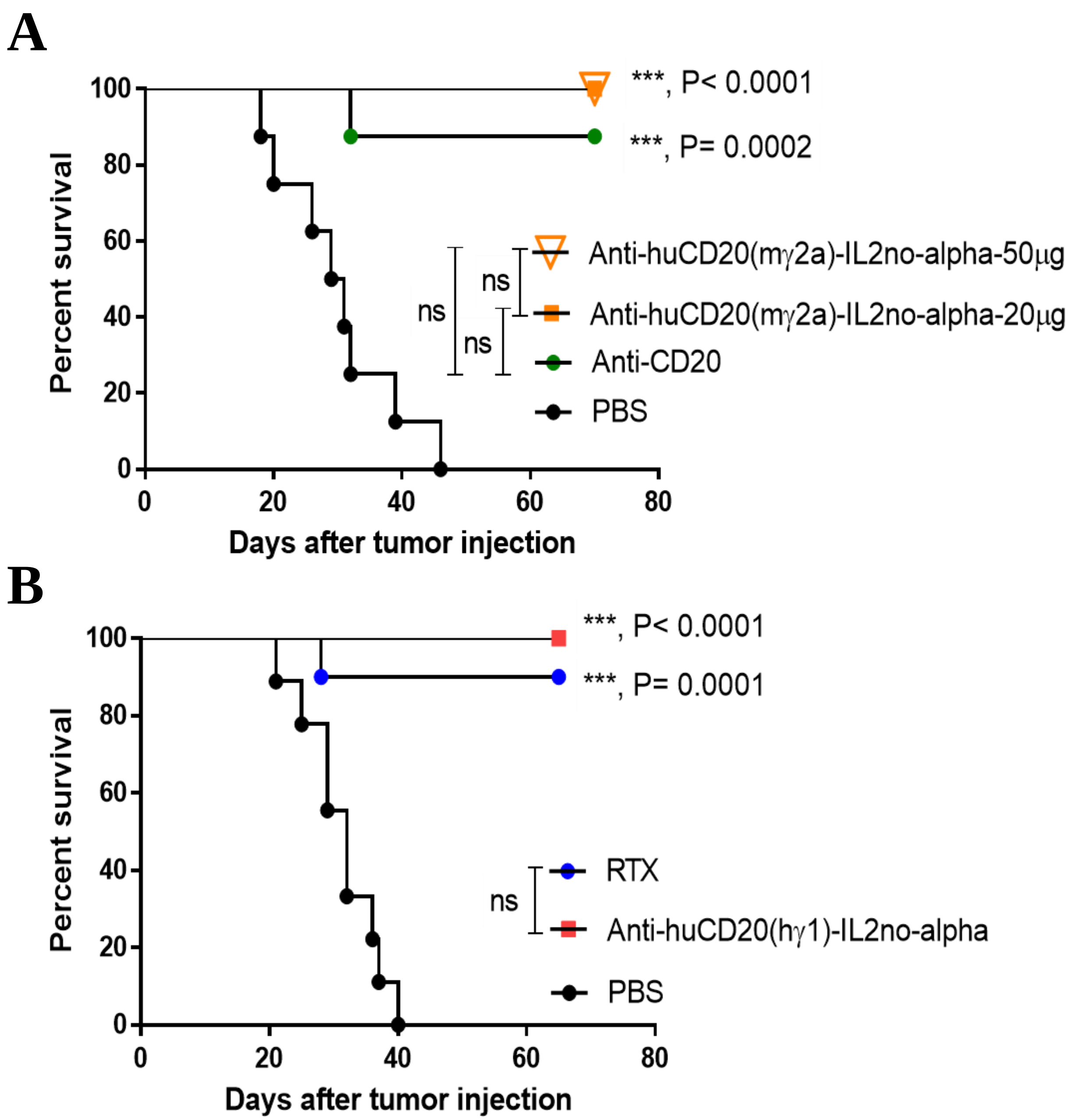

Supplementary Figure 6. Effect of anti-huCD20(mγ2a)-IL2no-alpha and anti-huCD20(hγ1)-IL2no-alpha ICK on survival of EL4-huCD20 tumor-bearing C57Bl/6 mice. **(A)** Survival curves of C57Bl/6 mice that were injected i.v. with EL4-huCD20+ cells and treated at Days 1, 4, 7,10, 14 with PBS, 200μg of anti-CD20, or 20 or 50μg of anti-huCD20(mγ2a)-IL2no-alpha ICK at days 1, 4 and 7. Data correspond to a representative experiment of two independent experiments (n=8 per group). (log-rank test; ***, P < 0.001 compared with PBS group; ns: not significant). **(B)** Survival curves of C57Bl/6 mice that were injected with EL4-huCD20 cells and treated at Days 1,4, and 7 with PBS, 150μg of RTX, or 20μg of anti-huCD20(hγ1)-IL2no-alpha ICK. Data correspond to a representative experiment of two independent experiments (PBS n=9; n=10 per group). (log-rank test; ***, P < 0.001 compared with PBS group; ns: not significant).

Supplementary Tables

Supplementary Table 1. Means and Medians for Survival Time (days) of **Figure 6B**

Treatment	Mean				Median			
	Estimate	Std. Error	95% Confidence Interval		Estimate	Std. Error	95% Confidence Interval	
			Lower Bound	Upper Bound			Lower Bound	Upper Bound
Anti-CD20	23,400	,980	21,480	25,320	25,000	1,265	22,521	27,479
Anti-CD20+IL2no-alpha	22,200	1,031	20,180	24,220	21,000	,775	19,482	22,518
Anti-huCD20(mγ2a)-IL2no-alpha	36,000	4,508	27,165	44,835	28,000	.	.	.
PBS	21,800	,917	20,004	23,596	20,000	2,372	15,351	24,649
Overall	25,850	1,516	22,879	28,821	25,000	,813	23,407	26,593

Supplementary Table 2. Means and Medians for Survival Time (days) of **Figure 6C**

Treatment	Mean				Median			
	Estimate	Std. Error	95% Confidence Interval		Estimate	Std. Error	95% Confidence Interval	
			Lower Bound	Upper Bound			Lower Bound	Upper Bound
Anti-huCD20(hγ1)-IL2no-alpha	54,500	5,125	44,455	64,545	.	.	.	.
PBS	29,000	2,814	23,485	34,515	27,000	2,981	21,156	32,844
RTX	37,900	5,830	26,473	49,327	27,000	5,270	16,670	37,330
Overall	40,862	3,415	34,168	47,556	35,000	1,345	32,363	37,637
